# Acute effect of whole-body vibration on hand grip strength, muscular activity, and upper limb function in young females with smartphone addiction: a randomized controlled trial

**DOI:** 10.3389/fmed.2026.1800879

**Published:** 2026-04-20

**Authors:** Nesma M. Allam, Ruyuf Abdullah Alkhaldi, Wadha Zaben Alshammari, Mohamed EL-Dosoky Mohamed Salama, Sally Yussef Abed, Khalid M. Ibraheem, Hasnaa Ali Ebrahim, Mohamed El-Sherbiny, Hadeel Essam Salem, Mohamed Mahmoud Abdelfattah Abdelrahman, Hadaya Mosaad Eladl

**Affiliations:** 1 Department of Physical Therapy for Surgery, Faculty of Physical Therapy, Cairo University, Giza, Egypt; 2 Department of Physical Therapy and Health Rehabilitation, College of Applied Medical Sciences, Jouf University, Sakaka, Saudi Arabia; 3 Department of Neuroscience Technology, College of Applied Medical Sciences in Jubail, Imam Abdulrahman Bin Faisal University, Jubail, Saudi Arabia; 4 Department of Respiratory Care, College of Applied Medical Sciences in Jubail, Imam Abdulrahman Bin Faisal University, Jubail, Saudi Arabia; 5 Department of Anesthesia Technology, College of Applied Medical Sciences in Jubail, Imam Abdulrahman Bin Faisal University, Jubail, Saudi Arabia; 6 Department of Basic Medical Sciences, College of Medicine, Princess Nourah bint Abdulrahman University, Riyadh, Saudi Arabia; 7 Department of Basic Medical Sciences, College of Medicine, AlMaarefa University, Diriyah, Saudi Arabia; 8 Department of Anaesthesia and Critical Care, King Abdulaziz University Hospital, King Abdulaziz University, Jeddah, Saudi Arabia; 9 Department of Anaesthesia and Surgical Intensive Care, Faculty of Medicine, Mansoura University, Mansoura, Egypt

**Keywords:** electromyography, hand grip strength, smartphone addiction, whole body vibration, young female

## Abstract

**Objective:**

To assess the efficacy of whole-body vibration (WBV) on hand grip strength, muscular activity, and upper limb function in young females with smartphone addiction.

**Materials and methods:**

This is a single-blinded randomized controlled trial. 66 females aged 18–25 years with smartphone addiction were randomly distributed into two equal groups; WBV group: It received WBV with strengthening exercises, and Sham WBV group: that received Sham WBV plus strengthening exercises. All participants received two sessions per week for four consecutive weeks. The primary outcome was hand grip strength. Secondary outcomes included pinch strength, muscle activity, and upper limb function. Evaluation was performed at baseline and after 4 weeks.

**Results:**

Post-treatment, there were significantly greater improvement in all variables with more favor to WBV group (*p* < 0.001) compared to the Sham WBV group. Mean differences (95% CI) between both groups were 9.48 [7.07, 11.88] for hand grip strength, which is the primary outcome.

**Conclusion:**

WBV combined with hand strengthening exercises might have a positive effect in enhancing hand grip strength, pinch strength, muscle activity, and upper limb function among young females with smartphone addiction. WBV could be a valuable adjunct to traditional rehabilitation programs targeting musculoskeletal effects of smartphone overuse.

**Clinical trial registration:**

ClinicalTrials.gov, identifier: NCT 06849687

## Introduction

Smartphones are considered a necessary part of everyday life, especially for young adults and adolescents (1). The increased usage of smartphones may be due to the rapid development of smartphone technologies, which include high-resolution touch screens, internet-based social networks, electronic banking, mobile gaming, and entertainment applications (2). The excessive and uncontrolled smartphone use is known as smartphone addiction, which has a negative impact on daily life, academic, and job performance (3). Smartphone addiction is becoming more prevalent internationally, especially among adolescents and young adults, which has raised significant concerns about its physical, behavioral, and psychological effects (4).

Smartphone addiction is related to a number of musculoskeletal issues, especially those that impact the upper limbs. Static and repetitive smartphone use combined with bad posture may reduce blood flow to the muscles. These problems can include ligament deterioration and muscular exhaustion in body parts such as the arms, wrist, fingers, shoulders, and neck that are extensively employed during using smartphones (5). Participating in smartphone activities without adequate elbow support and frequent thumb motions can exert excessive static pressures on the musculoskeletal and neural structures of the upper extremities (6).

Despite smartphones being designed to be used by two-handed, young adults frequently use it with one hand, predominantly the dominant hand. When using one hand, the thumb is primarily responsible for using keys, however, the remaining part of the hand provides support, leading to more weakness in the grip strength (7, 8). Studies conducted on young adults have reported that high-frequency smartphone users exhibit significantly lower hand grip strength, reduced pinch strength, and increased discomfort in the wrist and hand compared with individuals with lower smartphone usage (9–11). Recent evidence has also demonstrated that prolonged smartphone usage has been associated with decreased hand performance and impaired upper limb function due to repetitive strain and overuse of intrinsic hand muscles (12).

Whole-body vibration (WBV) training has emerged as a promising safe and effective exercise modality in rehabilitation for enhancing performance, and muscular activity. It involves standing or supporting the body on a vibrating platform that generates mechanical oscillations with varying frequencies and amplitudes (13). These oscillations stimulate the neuromuscular system, and activate muscle spindles, leading to reflexive muscle contractions known as the tonic vibration reflex (TVR), that may facilitate muscle activation and motor unit recruitment (14). Additionally, WBV has been shown to improve strength, muscle activation, and functional performance (15).

Previous studies have evaluated the impact of WBV in enhancing hand grip strength, muscular activity, and functional performance in different populations, including patients with rheumatoid arthritis (RA), stroke, athletes, healthy adults, and elderly (15–19). However, the possible therapeutic impact of WBV in treating upper limb deficits caused by overuse of smartphones has received very limited focus. To the best of the authors' knowledge, no previous study has been evaluated the influence of WBV training on the neuromuscular performance of upper limbs in young females with smartphone addiction. Therefore, there is a lack in the present literature about the efficacy of WBV training as a therapeutic approach to enhance neuromuscular performance in females with excessive smartphone use. Addressing this gap is especially crucial since young females are among the most frequent users of smartphones and may be more prone to smartphone addiction and musculoskeletal issues in the upper limb due to the hormonal factors, and differences in muscle strength when compared to males (20, 21). Finding efficient treatments that can improve upper limb function and neuromuscular performance in this population may help avoid long-term musculoskeletal complications related to excessive smartphone use. Thus, the purpose of the current study was to detect the impact of WBV on muscular activity, hand grip strength, and upper limb function in young females with smartphone addiction. The authors hypothesized that WBV training in addition to strengthening exercises would have significant improvements in hand grip strength, muscular activity, and upper limb function compared with those who received the strengthening exercises alone.

## Materials and methods

### Study design

The current study is a single-blinded (Assessor only), randomized sham-controlled trial, performed between April and September 2025 at the Physical Therapy and Health Rehabilitation laboratory, College of Applied Medical Sciences, Jouf University. The present study was carried out based on the Declaration of Helsinki standards. It was approved by the Ethical Committee for Scientific Research, Jouf University (N0: 7486), and prospectively registered at Clinical Trials.gov (NCT 06849687). Participants were selected from College of Applied Medical Sciences, Jouf University. All participants received a detailed explanation of the procedure's steps and objectives before participation, and they were permitted to withdraw from the trial at any time, followed by obtaining written informed consent from every participant. This study followed CONSORT guidelines.

### Participants

Participants were selected for the current study by the second author (R.A.A.), who was not involved in applying the intervention or measuring outcome measures to decrease bias, according to the following inclusion criteria: female participants in order to minimize the confounding consequences associated with sex differences in smartphone using behaviors and musculoskeletal characteristics, between the ages of 18 and 25 years old, classified as high smartphone users according to the score of the Arabic version of Smartphone Addiction Scale Short Version (SAS-SV) >33 (22), using smartphone predominantly with their dominant hand for at least 5 h/day during the last 6 months; as they had significantly weaker grip strength (8, 23); with upper limb dysfunction due to prolonged smartphone use with a cut-off value of more than 10 in the Quick Disabilities of the Arm, Shoulders, and Hand (DASH) questionnaire that indicate clinically relevant disability of the upper limb for females in this age group (24), with a body mass index (BMI) of < 30 kg/m^2^, not engaged in regular exercise over the past 6 months, with the ability to adhere to the directions and complete the required assessments. The exclusion criteria were participants with any peripheral nerve injury or surgical release in the upper limb (e.g., median, ulnar, or radial nerve lesions), any musculoskeletal disorders (such as; tendon lesions, prior bone fractures of the wrist or hand), or skin diseases affecting the upper limb, acute thrombosis, tumors, recent implants, extremely delayed onset of arm and trunk muscle soreness, as well as peripheral vascular disease. Additionally, individuals with contraindications to WBV, such as acute inflammation, injuries to the back, joint problems, seizure disorders, diabetes, or neuromuscular diseases (25).

### Sample size

The sample size was detected *a priori* using G^*^Power software (version 3.1, Universität Düsseldorf, Germany). The calculation was based on the primary outcome of hand grip strength, which was analyzed using a repeated-measures MANOVA, testing the group × time interaction. Type I error rate (α) = 0.05, effect size = 0.38 Ahn et al. (26), and statistical power (1–β) = 0.80. Under these assumptions, G^*^Power indicated that a minimum of 57 participants would be required to detect a significant within–between interaction. To account for possible attrition and missing data, the required sample was inflated by 15%, resulting in a final target of 66 participants (33 per group).

### Randomization

Sixty-six participants with smart phone addiction were randomly distributed to either the WBV group or the Sham WBV group. Block randomization was conducted, by a research assistant who was not included in the assessment, data collection or intervention procedures, utilizing a computer-based tool with the allocation ratio of 1:1 and blocks of four, six, and eight to reduce bias. The randomization process was conducted before starting assessment of participants. The evaluator responsible for data collection remained blind to group assignments throughout the experiment. To ensure allocation concealment, randomization codes were sequentially labeled and securely stored in opaque, sealed envelopes.

### Intervention

After the termination of baseline assessments, the first author (N.M.A.) unsealed closed envelopes including the group allocations of participants. Participants were randomly distributed between two groups: the study group (WBV group), who received WBV, in addition to strengthening exercises, and the control group (sham WBV group), who performed the same WBV Program but without mechanical vibration, in addition to strengthening exercises. A familiarization session was conducted for all participants to be familiar with WBV device, strengthening exercises, and procedures performed in subsequent experimental conditions (18). Every session started with a 10-min warm-up, including stretches for the wrist and finger flexors and extensors, in addition to range of motion (ROM) exercises for the wrist and fingers (27). The intervention was performed two sessions per week for 4 weeks. By asking participants which hand they use for writing, the dominant hand was detected.

### Exercise program

#### Whole body vibration (WBV)

WBV training was conducted using a vibratory platform (Power Plate Pro7HC, Northbrook, IL, USA), with the device turned on for the WBV group. Vibration parameters were set at a frequency of 45 Hz and an amplitude of 2 mm, based on previous studies (14, 18). Participants assumed a modified push-up position on the vibration platform, with their hands placed 28 cm apart and elbows flexed at 10 degrees. The modified push-up position was selected as it was proved that it increased upper limb muscle activation following acute WBV stimulation (16, 18). Each WBV session is composed of 20 bouts, with each bout including 10 s of vibration followed by 10 s of rest. After the first 10 bouts, a 4-min pause was implemented before continuing, following previously established protocol (28). The total procedures lasted about 10 min. If the participant experienced nausea or dizziness during the procedures, we immediately ceased the trial, and the participant was placed in a supine position.

Participants in the Sham WBV group were placed on the same vibration platform and adhered to the same guidelines for position, duration, and session protocols as those in the WBV group. No mechanical vibration was produced, but the device was turned on to preserve uniformity in visual signals (such as the display panel and indication lights). To reduce the likelihood of participants recognizing the absence of vibration, some strategies were used to prevent participants from being unblinded, such as selecting participants who were new to WBV therapy, treating and evaluating them on different days so that there was never any communication between the participants in both groups. Additionally, participants were told that WBV would not cause them to have any kind of sensation. In order to help deflect attention from vibration input, they were also told to concentrate on keeping the necessary posture during the session.

#### Strengthening exercise

Participants in both groups performed strengthening exercises for their dominant hand utilizing a hand gripper. It was modified to 75% of each participant's gripping force, based on previous evaluation. Participants were instructed to sit on a chair supporting their backs and rested the dominant arm on a table with shoulder comfortably adducted, elbow flexion 90 °, forearm and wrist in neutral position. They were asked to press the adjustable handles by using their dominant hands until both contacted, holding the contraction for 4 s, then releasing for 2 s before repeating the movement continuously for 1 min. This exercise was performed in three sets of 1 min, with a 3-min rest in-between (29).

For finger extension and flexion exercises, elastic resistance was utilized (TheraBand Hand Xtrainer, and TheraBand Hand Exerciser respectively, TheraBand, Akron, OH, USA). The exercises were done in the full available ROM for each participant from sitting position with the forearm rested on the table. The participants were told to perform two exercises against resistance: flexion exercise (squeezing the ball and releasing) and extension exercise (extending the fingers and releasing). Each exercise was performed in two sets of 20 repetitions, with each set consisting of 10 controlled movements (30).

### Outcome measures

The primary outcome was hand grip strength. Secondary outcomes included pinch strength, muscle activity, and upper limb function. All outcome measures were recorded at baseline and after 4 weeks by the third author (W.Z.A.), who was blinded to the group allocation. Participants were instructed to take rest for 15 min after each test, by placing their hands in supine position on their thighs.

#### Hand grip strength

Measurement of hand grip strength was taken under standardized conditions using (JAMAR^®^, Chicago, IL, USA) hydraulic hand-held dynamometer. It has a high intra-rater reliability, the Intraclass Correlation Coefficient (ICC = 0.98) (31). Participants sat on a chair maintaining adduction and neutral rotation of their shoulders, 90 ° elbow flexion, neutral position for forearm, 30 ° wrist extension, and 10 ° ulnar deviation. The dynamometer handle was modified to the setting suitable for the participant's hand. After receiving an illustration of the procedure and becoming familiar with the dynamometer, participants were instructed to perform maximum hand grip strength for 3–5 s with verbal encouragement. The test was repeated three times for dominant hand, with 1 min rest interval in between, then the mean value in Kg was recorded (32).

#### Pinch strength

Measurements were taken using (JAMAR^®^, Chicago, IL, USA) pinch dynamometer. It has high intra-rater reliability (ICC = 0.98) (31). Participants were asked to sit on a chair with their backs straight maintaining the same posture used for grip strength measurement. The examiner placed the pinch gauge on its side at a 45-degree angle while providing little support. It was positioned between the thumb's pulp and the middle and index fingers. For 3–5 s, participants were told to press the dynamometer as firmly as they could, then relax. There was a 1-min break between each of the three trials. The mean of the three trials in kilograms was recorded (33).

### Electromyography (EMG)

Muscle activity was measured using a Bagnoli-16 EMG (TrigoTM Wireless System, Delsys Inc., Boston, MA, USA) device, measuring root mean square (RMS). Double differential surface electrodes (DE-3.1) were utilized for recording. Before the experiment, each participant received brief training to ensure correct task execution. Before electrode placement, the skin was prepared by disinfecting with an alcohol-soaked cotton swab, shaving, and disinfecting again to decrease skin impedance. Electrodes were also cleaned with alcohol and secured with double-sided adhesive tape to ensure proper contact with the skin throughout the experiment (34).

During measurements, participants sat with their dominant upper limb resting on a table with the shoulder comfortably positioned in abduction 30 °, the hand in a neutral orientation, and elbow flexed 90 °. Participants were asked to conduct isometric maximum voluntary contractions (MVCs) for flexion/extension for 5 s, with 10 s rest in between, and a 5-min rest after each test (35). EMG signals were recorded from the extensor digitorum communis (EDC), flexor carpi radialis (FCR), and flexor carpi ulnaris (FCU) muscles. Those muscles were selected because of their importance to wrist and hand function throughout handgrip. Electrodes were applied on the muscle belly along the muscle fibers (34).

Raw EMG data were sampled at 4,000 Hz, pre-amplified near the electrodes (signal bandwidth of 20–450 Hz), and saved on a laptop. A special software (Delsys EMGworks Analysis 4.0. Delsys Inc. Boston, MA, USA) was used to analyze the EMG data and determine the RMS. Peak to peak hand strength values were used to standardize the EMG readings for every test condition. The mean of thre repetitions completed both prior to and after the intervention procedures was used to determine EMGrms (18).

### Quick disabilities of the arm, shoulders, and hand (DASH)

The 11-item Arabic QuickDASH is a patient-reported outcome measure that measures upper extremity disorders' symptoms and activity limitations. Every item was given a value between 1 and 5, where 1 denotes no functional limitations or symptoms and 5 denotes severe symptoms and functional incapacity. A higher score indicates more activity constraint and worse symptoms. The overall score goes from 0 to 100. With an ICC of 0.91, the Arabic version exhibits high internal consistency and test-retest reliability (36).

### Statistical analysis

Statistical analyses were carried out using SPSS software (version 25; SPSS Inc., Chicago, IL, USA), with the significance level established at 0.05. Prior to the main analyses, preliminary checks were conducted to ensure that key assumptions were met, including normality, homogeneity of variances, and the absence of significant outliers. The Shapiro-Wilk test was applied to evaluate the normal distribution of continuous variables (*p* > 0.05). For multivariate analyses, additional assumptions were assessed: Box's *M* test was used to examine the equality of covariance matrices, and Pearson correlation coefficients were calculated to detect any multicollinearity among the dependent variables.

Assumptions were considered sufficiently satisfied. While overall model fit indicators are not typically used in MANOVA, residual plots for each dependent variable were visually examined to evaluate symmetry in distribution, identify any outliers, and assess the consistency of variance. The residuals showed an approximately normal distribution and were evenly spread, with no major assumption violations observed. Data were presented as mean ± standard deviation for continuous variables and as counts for categorical variables such as Dominance. To manage missing values during the 4-week intervention, an intention-to-treat analysis was implemented using multiple imputation techniques. A two-way mixed-design MANOVA was conducted to evaluate the combined effects across multiple outcome variables, which was specifically chosen because the study included several linked dependent variables assessed throughout two groups and two time points. The outcomes evaluated reflect various but interrelated aspects of neuromuscular performance and upper limb function. Additionally, MANOVA assesses group effects, time effects, and group × time interactions throughout all dependent variables in a single multivariate model, while taking into account the correlation structure between them. Where MANOVA showed significant results, individual follow-up ANOVAs with Bonferroni adjustment were performed to minimize type I error.

## Results

**Figure 1:**
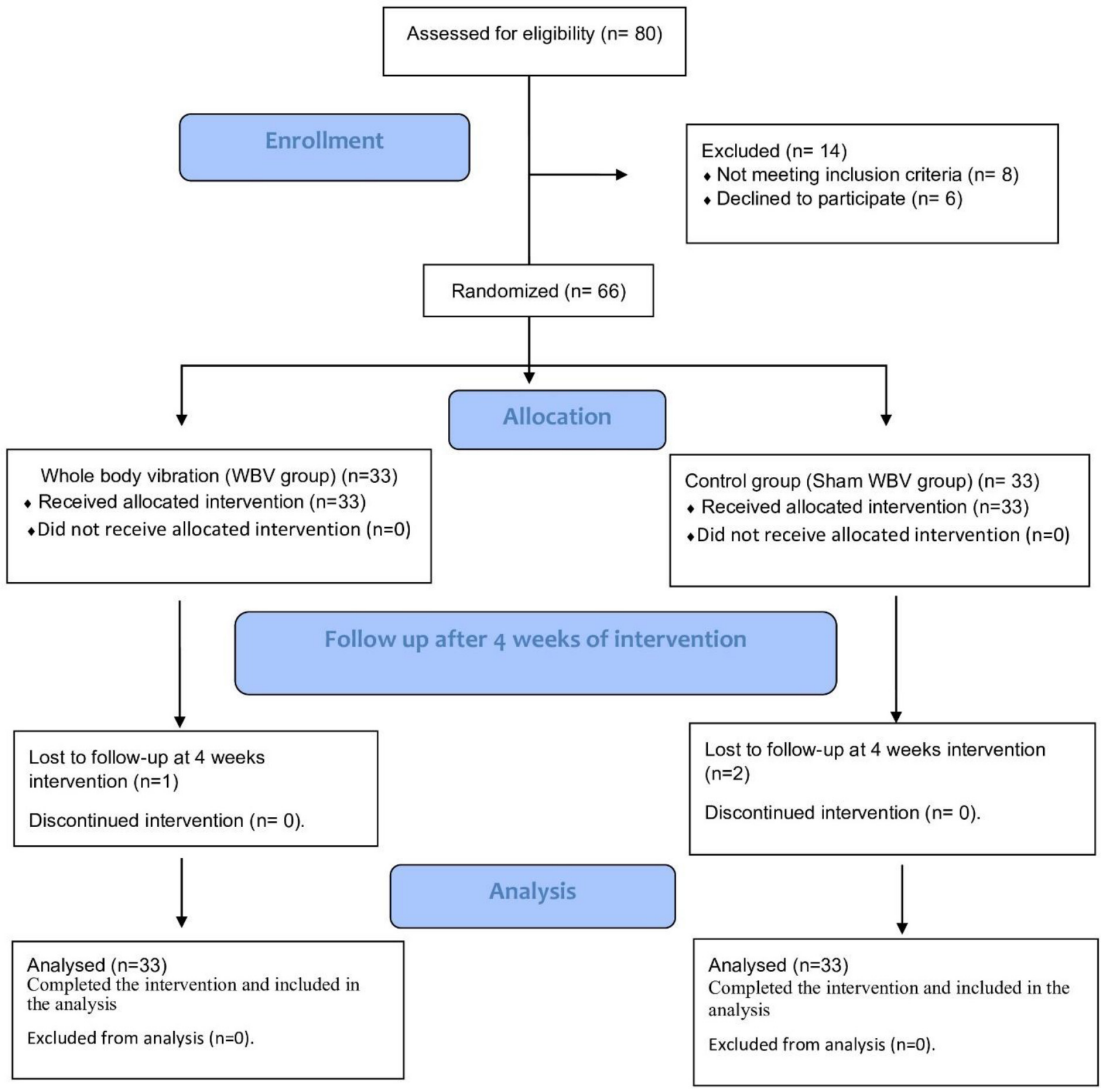
Flow chart of participants.

Figure 1 illustrates the study flow. According to the CONSORT diagram, 3 of the 66 participants (4.5%) did not complete the post-treatment evaluation (1 belonging to the WBV group and 2 to the Sham WBV group). To address the missing information, a regression-based multiple imputation method was applied, generating three datasets as recommended for studies with minimal missingness.

There were no significant differences between the groups at baseline in terms of demographic characteristics, including age, weight, height, BMI, daily usage duration, and addiction scores (*p* > 0.05; Table 1). Likewise, the distribution of hand dominance showed no significant variation between the groups, χ^2^ (1, *N* = 66) = 0.41, *p* = 0.52. Baseline comparisons also revealed no statistically significant differences in hand grip strength, pinch grip strength, DASH scores, or EMG activity of the FCR, FCU, and EDC muscles between the groups (*p* > 0.05; Table 2).

**Table 1:** Baseline demographic characteristics of participants (*N* = 66)^*^.

Characteristics	WBV group (*n* = 33)	Sham WBV group (*n* = 33)	MD (95% CI)	*p*-value
Age (years)	21.33 ± 1.98	21.52 ± 2.03	−0.18 (−1.17, 0.80)	0.71
Weight (kg)	59.24 ± 7.07	60.58 ± 7.10	−1.33 (−4.81, 2.15)	0.45
Height (cm)	158.09 ± 5.93	157.18 ± 9.31	0.91 (−2.93, 4.75)	0.64
BMI (kg/m^2^)	23.96 ± 2.43	24.49 ± 2.40	−0.53 (−1.72, 0.66)	0.38
Usage (hours)	11.42 ± 2.15	11.61 ± 1.95	−0.18 (−1.19, 0.83)	0.72
Addiction	41.88 ± 6.69	42.24 ± 6.69	−0.63 (−3.66, 2.92)	0.81
Dominance, *n* (%)				
Right	28 (84.8%)	26 (78.8%)	*X*^2^ = 0.41	0.52
Left	5 (15.2%)	7 (21.2%)		

BMI, body mass index; X^2^, chi square; MD, mean difference; Kg, kilogram; CI, confidence interval. ^*^Data are mean ± SD for all demographics except dominance (%), p-value < 0.05 indicate statistical significance.

**Table 2:** The baseline clinical characteristics of all outcome variables (*N* = 66)^*^.

Outcomes	WBV group (*n* = 33)	Sham WBV group (*n* = 33)	MD (95% CI)	*p*-value
Hand grip strength (kg)	16.92 ± 4.76	17.10 ± 3.75	−0.19 (−2.29, 1.92)	0.86
Pinch grip strength (kg)	5.36 ± 1.59	5.51 ± 1.82	−0.16 (−1.0, 0.67)	0.71
DASH (%)	39.07 ± 14.31	38.27 ± 14.60	−0.81 (−7.92, 6.30)	0.82
Electromyography root mean square (EMG RMS)				
Flexor carpi radialis (mV)	4.76 ± 1.37	4.70 ± 1.61	0.06 (−0.68, 0.79)	0.88
Flexor carpi ulnaris (mV)	4.93 ± 1.37	5.06 ± 1.32	−0.12 (−0.79, 0.54)	0.71
Extensor digitorum communis (mV)	4.36 ± 1.56	4.44 ± 1.34	−0.08 (−0.79, 0.64)	0.83

DASH, the disabilities of the arm, shoulder and hand; Kg, kilogram; mV, millivolt; MD, mean difference; CI, confidence interval.

^*^Data are mean ± SD, p-value < 0.05 indicate statistical significance.

A mixed-design MANOVA was employed to evaluate differences in the extent of change across outcome measures between the two groups. The analysis revealed significant multivariate effects for the main effect of group, Wilk's λ = 0.6, *F*_(6, 59)_ = 6.63, *p* < 0.001, η^2^ = 0.4; for time, Wilk's λ = 0.03, *F*_(6, 59)_ = 367.1, *p* < 0.001, η^2^ = 0.97; and for the interaction between group and time, Wilk's λ = 0.06, *F*_(6, 59)_ = 149.8, *p* < 0.001, η^2^ = 0.94.

### Between-group comparisons

**Figure 2:**
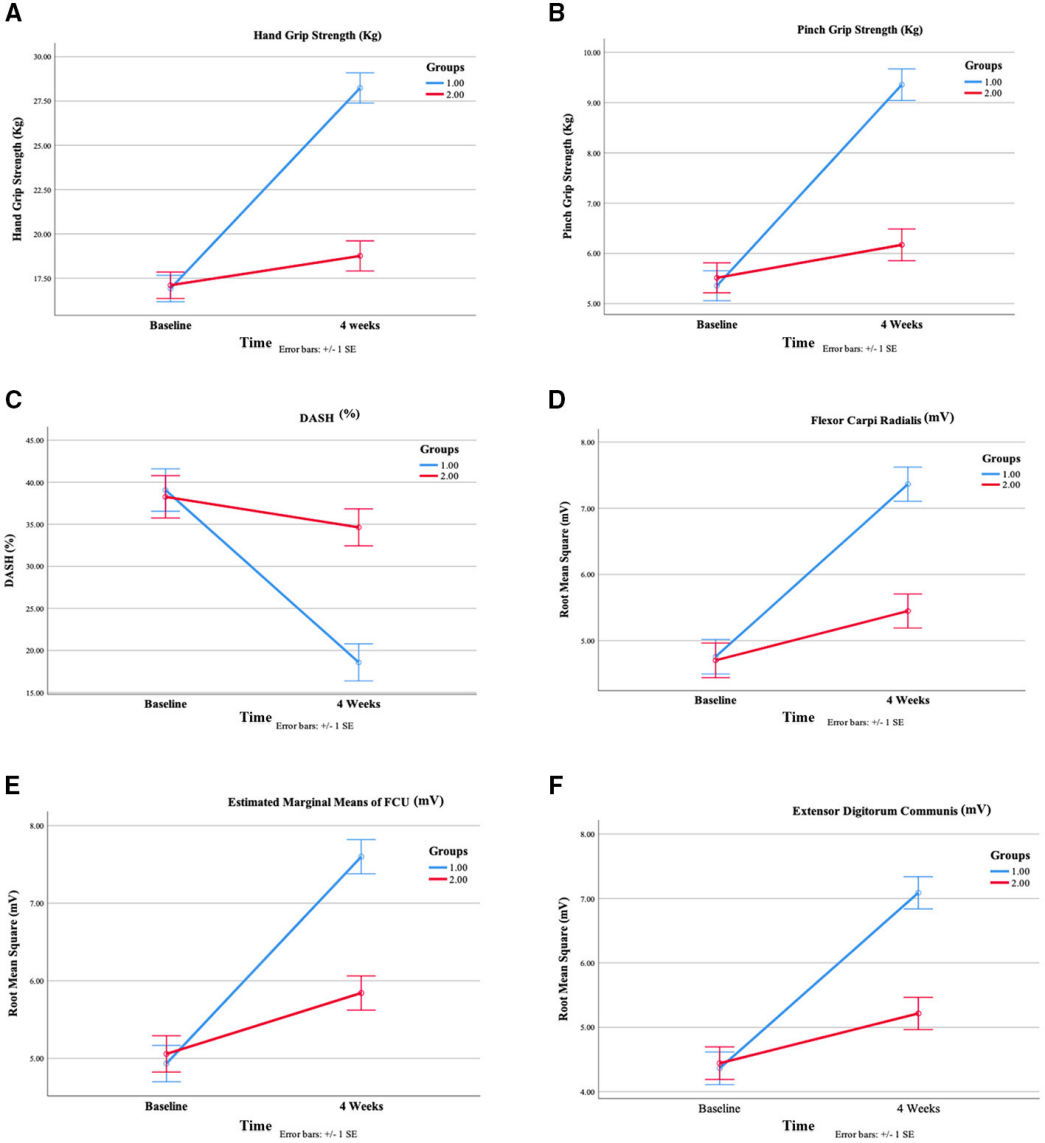
Estimated marginal means in both group for **(A)** hand grip strength **(B)** pinch grip strength **(C)** DASH **(D)** flexor carpi radialis RMS **(E)** flexor carpi ulnaris RMS **(F)** extensor digitorum communis.

Following the 4-week intervention period, notable differences between the groups were identified in all primary and secondary outcomes (Table 3 and Figure 2). Participants in the WBV group showed significantly greater enhancement in hand grip strength (M = 28.34, SD = 5.88) than those in the Sham WBV group (M = 18.76, SD = 3.63), with a mean difference of 9.48, 95% CI [7.07, 11.88], *p* < 0.001, η^2^ = 0.49. Likewise, improvements in pinch grip strength were more pronounced in the WBV group (M = 9.36, SD = 1.80) compared to the Sham WBV group (M = 6.17, SD = 1.81), yielding a mean difference of 3.19, 95% CI [2.30, 4.08], *p* < 0.001, η^2^ = 0.44.

**Table 3:** Clinical characteristics of participants after 4 weeks of intervention for all outcome variables (*N* = 66)^*^.

Outcomes	WBV group (*n* = 33)	Sham WBV group (*n* = 33)	MD (95% CI)	*p*-value	Partial eta squared
Hand grip strength (kg)	28.34 ± 5.88	18.76 ± 3.63	9.48 (7.07, 11.88)	<0.001	0.49
Pinch grip strength (kg)	9.36 ± 1.80	6.17 ± 1.81	3.19 (2.30, 4.08)	<0.001	0.44
DASH (%)	18.58 ± 9.82	34.64 ± 14.92	−16.06 (−22.27, −9.84)	<0.001	0.29
Electromyography root mean square (EMG RMS)					
Flexor carpi radialis (mV)	7.36 ± 1.38	5.45 ± 1.58	1.92 (1.19, 2.64)	<0.001	0.31
Flexor carpi ulnaris (mV)	7.60 ± 1.12	5.84 ± 1.40	1.76 (1.14, 2.38)	<0.001	0.33
Extensor digitorum communis (mV)	7.09 ± 1.58	5.22 ± 1.28	1.87 (1.17, 2.58)	<0.001	0.30

DASH, the disabilities of the arm, shoulder and hand; Kg, kilogram; mV, millivolt; MD, mean difference; CI, confidence interval.

^*^Data are mean± SD, p-value < 0.05 indicate statistical significance.

The WBV group also exhibited significantly greater improvement in functional outcome, reflected by DASH scores (M = 18.58, SD = 9.82), compared to the Sham WBV group (M = 34.64, SD = 14.92). The mean difference was −16.06, with a 95% CI [-22.27, −9.84], *p* < 0.001, η^2^ = 0.29.

Regarding muscular activation, the WBV group experienced significantly greater increases in EMG RMS values compared to the Sham WBV group. These increases were observed in the FCR (mean difference = 1.92 mV, *p* < 0.001, η^2^ = 0.31), FCU (mean difference = 1.76 mV, *p* < 0.001, η^2^ = 0.33), and EDC muscles (mean difference = 1.87 mV, *p* < 0.001, η^2^ = 0.30).

### Within-group comparisons

Within-group analyses confirmed that the WBV group exhibited substantial improvements in all outcome measures from baseline to post-intervention. Specifically, large increases were noted in hand grip strength [mean change = −11.32, 95% CI (−12.43, −10.20), *p* < 0.001] and pinch grip strength [mean change = −4.00, 95% CI (−4.29, −3.71), *p* < 0.001]; as negative change values demonstrate enhancement, indicating an increase in muscle strength from baseline to 4 weeks; as well as significant reductions in DASH scores [mean change = 20.49, 95% CI (18.49, 22.48), *p* < 0.001]; as positive change values display improvement, representing a reduction in disability. Large increases were also noted in FCR RMS [mean change = −2.61, 95% CI (−2.89, −2.33), *p* < 0.001] and FCU RMS [mean change = −2.67, 95% CI (−2.86, −2.47), *p* < 0.001], as well as in EDC RMS [mean change = −2.72, 95% CI (−2.97, −2.48), *p* < 0.001]; as negative change values indicate improvement, demonstrating an increase in muscle activation from baseline to 4 weeks. In contrast, although the Sham WBV group also showed improvements, the magnitude of change was smaller across all measures (Table 4).

**Table 4:** Within-groups comparisons for all outcome measures.

Outcomes	WBV group (*n* = 33)		Sham WBV group (*n* = 33)	
	Change from baseline to 4 weeks		Change from baseline to 4 weeks	
	MD (95% CI)^*^	*p*-value	MD (95% CI)^*^	*p*-value
Hand grip strength (kg)	−11.32 (−12.43, −10.20)	< 0.001	−1.66 (−2.77, −0.54)	0.004
Pinch grip strength (kg)	−4.00 (−4.29, −3.71)	<0.001	−0.66 (−0.95, −0.37)	<0.001
DASH (%)	20.49 (18.49, 22.48)	<0.001	3.63 (1.63, 5.62)	0.001
Electromyography root mean square (EMG RMS)				
Flexor carpi radialis (mV)	−2.61 (−2.89, −2.33)	<0.001	−0.75 (−1.03, −0.47)	<0.001
Flexor carpi ulnaris (mV)	−2.67 (−2.86, −2.47)	<0.001	−0.79 (−0.98, −0.59)	<0.001
Extensor digitorum communis (mV)	−2.72 (−2.97, −2.48)	<0.001	−0.77 (−1.02, −0.53)	<0.001

DASH, the Disabilities of the Arm, Shoulder and Hand; Kg, kilogram; mV, millivolt; MD, mean difference; CI, confidence interval.

^*^Data are MD (95% CI), *P*-value < 0.05 indicate statistical significance.

Change values were calculated as (baseline − 4 weeks). Negative change values for hand grip strength, pinch grip strength, and EMG RMS represent improvements, whereas positive change values for DASH represent improvements.

## Discussion

The present study assessed the effects of WBV in conjunction with strengthening exercises on hand grip strength, pinch strength, muscle activity, and upper limb function in young females with smartphone addiction. After 4 weeks of intervention, participants in the WBV group reported significantly greater improvements in all evaluated outcomes (*p* < 0.001) than those in the Sham WBV group. These findings revealed that WBV can be a useful supplemental intervention when combined with traditional hand strengthening exercises to improve upper limb performance in young females with excessive smartphone use.

WBV group exhibited a significant improvement in grip strength, and pinch strength (*p* < 0.001) in comparison to the Sham WBV group, which exceeded the minimum clinically important difference (MCID) that was 2.69 kg for grip strength as well as pinch strength that was 0.68 kg for the dominant hand in healthy population (37). The mechanism underlying the advantageous effects of WBV on grip, and pinch strength may be attributed to activation of neuromuscular system by the vibration stimuli which is transferred through sensory receptors. A complicated spinal and supraspinal neurophysiological response known as the tonic vibration reflex (TVR) is triggered by the transmission of this mechanical oscillation. In turn, this TVR can activate muscles and enhance physical performance (16, 19). These improvements are functionally important and likely to translate into improved performance in daily activities involving manual dexterity and grip function and may help avoiding musculoskeletal complications linked to smartphone overuse.

These results are consistent with previous studies that reported beneficial effects of WBV on strength of hand muscles (14, 16–18, 38, 39). Some studies used the same vibration frequency, amplitude and the modified push up position used in the current study and found a positive acute impact of WBV on strength of hand muscles in females suffering from RA (16), and healthy adults (14, 18). A systematic review and meta-analysis by Zeng et al. (17) demonstrated positive effect of WBV training on muscle strength in patients with stroke. Similarly, it has been reported that WBV improved muscle strength of the upper limb in children with hemiplegia (38) and improve muscle quality in elderly with osteosarcopenia (39). However, some of these studies were applied to other populations with varying age groups, vibration parameters, and intervention duration. The current study contributes the body of evidence by revealing that WBV combined with hand strengthening exercises can enhance hand grip strength in young females with smartphone overuse, highlighting both statistically and clinically significant effect. Contradictory findings were observed in some previous studies on healthy and older adults (19, 29). This contradiction may be explained by variations in lower vibration frequency, different duration, posture, and population characteristics which may restrict the neuromuscular stimulation important to produce strength improvements.

The present study also demonstrated a significant increase in EMG activity in the WBV group for the forearm muscles (FCR, FCU, and EDC; *p* < 0.001) when compared with the Sham WBV group, which indicates that WBV enhances motor unit recruitment beyond the effects of strengthening exercises alone (18). Additionally, it has been demonstrated that WBV affects cortical and subcortical motor regions, which modulate central motor drive and enhance motor control. Higher EMG amplitudes indicate more effective firing of motor units, synchronization, that lead to greater muscle recruitment throughout voluntary contraction (40, 41). Clinically, these alterations lead to increased force output, enhanced muscle coordination, and better control while moving hand and wrist, which may result in improved motor function and lower risk of overuse injuries. These results proof to the use of WBV as a functional neuromuscular therapy in young females suffering from musculoskeletal issues due to smartphone overuse.

The findings of the present study are supported by other studies (16, 28) demonstrating that vibration exposure can increase muscle activation. These studies used the same high-frequency vibration as that is used in our study as this frequency resulted in the highest level of muscle activation as compared with low-frequency vibration based on the EMGrms (16, 28). Additionally, it was found that acute WBV applied directly beneath the hands from the modified push-up position enhances EMGrms activity in wrist muscles among women with stable RA (16). Comparable results have also been found in studies evaluating lower limb muscles (42, 43) who reported a significant effect of WBV on enhancing EMG activity in lower limb muscles in other populations, indicating that oscillation stimuli can improve neuromuscular activation whatever the muscle being targeted. Additionally, Yoon et al. (44) found that even low-frequency WBV can increase muscle activation in healthy adults. Although these similarities, variations in population, vibration frequency, targeted muscle groups and duration of intervention in some studies may affect the strength of EMG responses. These comparisons reveal that WBV frequency, body position over the platform, and population characteristics can affect the intensity and nature of EMG response, with higher frequencies and suitable positioning are expected to result in higher neuromuscular facilitation, as observed in the current study.

The present study also revealed a statistically significant improvement in upper limb function in the WBV group compared to the Sham WBV group (*p* < 0.001). These observed changes are not only statistically significant but also meaningful to participants' manual performance and quality of life as it exceeded the MCID for upper limb function that is ranged from 12 to 15 points for Quick DASH in people with musculoskeletal disorders (45). This enhancement can be attributed to stimulation of sensorimotor integration within the central nervous system, which may improve the accuracy of upper limb movements and enable greater performance throughout functional tasks (16). Furthermore, vibration stimuli may enhance muscle perfusion, and local blood supply, which would enhance oxygen delivery and metabolism in the forearm muscles. These physiological changes can enhance muscular endurance and whole functional performance of the upper limb (46). From a clinical perspective, this improvement implies that participants' capacity to carry out everyday tasks requiring hand and wrist function such as gripping, handling objects, and carrying out repetitive hand use tasks was noticeably improved. This is particularly important for subjects who use smartphone excessively, as extended repeated hand activity can cause discomfort and decreased upper limb function.

Previous studies have supported the positive effect of WBV when combined with traditional exercises in enhancing upper limb function across different populations (19, 26, 47, 48). A recent systematic review and meta-analysis by Lu et al. (47) and randomized controlled trials by Ahn et al. (26) and Lee et al. (48) demonstrated that WBV significantly enhances upper limb function when combined with conventional exercises in patients with stroke. Additionally, enhancements in functional performance after WBV training have also been found in older adults (19). Despite these studies were performed in populations suffering from neurological deficits, various age groups and vibration parameters, the present findings extend this evidence by revealing that WBV can also enhance upper limb function in young females with musculoskeletal problems associated with excessive smartphone use. In contrast to our results, a pilot study by Kelaiditi et al. (49) found no statistically significant differences between groups in children with cerebral palsy. The discrepancy may be resulted from alterations in study design, vibration parameters, body position, sample size and shorter intervention duration, which may affect the magnitude of neuromuscular responses to WBV.

### Limitations and recommendations

This study has several limitations. First, the sample included only young females aged 18–25, using smartphone with their dominant hand restricting the generalizability of findings to males, other age groups, and individuals who use smartphones with both hands. Second, the 4-week intervention period and lack of follow-up may not reflect the longer-term effect of the training. Third, the single–blind design with only the assessor blinded as complete blinding in the Sham WBV group was challenging due to the perceptible nature of vibration. Fourth, the protocol of strengthening exercises and vibration parameters do not reflect the potential effects of varied sets, frequencies, or durations. Future research should include more diverse samples across gender and age groups, two hand users of smartphones, adopt double–blind designs when feasible, extend the intervention duration, and explore different sets, durations, of strengthening exercise training, and different WBV protocols. Future studies should incorporate follow-up evaluations to better assess the longevity of outcomes.

## Conclusion

A 4-week WBV program, particularly when combined with strengthening exercises, might improve hand grip strength, pinch strength, muscle activity (FCU, EDC, FCR), and upper limb function in young females with smartphone addiction. These findings highlight WBV as a tool to address musculoskeletal issues from excessive smartphone use and support its integration into physiotherapy for a more comprehensive rehabilitation approach.

## Data Availability

The original contributions presented in the study are included in the article/supplementary material, further inquiries can be directed to the corresponding author.

## References

[1] Nikolic A Bukurov B Kocic I Vukovic M Ladjevic N Vrhovac M . Smartphone addiction, sleep quality, depression, anxiety, and stress among medical students. Front Public Health (2023) 11:1252371. doi: 10.3389/fpubh.2023.1252371 37744504 PMC10512032

[2] Montag C Wegmann E Sariyska R Demetrovics Z Brand M. How to overcome taxonomical problems in the study of internet use disorders and what to do with “smartphone addiction”? J Behav Addict. (2021) 9:908–14. doi: 10.1556/2006.8.2019.59 31668089 PMC8969715

[3] Liu H Zhou Z Zhu E Huang L Zhang M. Smartphone addiction and its associated factors among freshmen medical students in China: a cross-sectional study. BMC Psychiatry (2022) 22:308. doi: 10.1186/s12888-022-03957-5 35501728 PMC9058751

[4] Salari N Zarei H Hosseinian-Far A Rasoulpoor S Shohaimi S Mohammadi M. The global prevalence of social media addiction among university students: a systematic review and meta-analysis. J Public Health (2025) 33:223–36. doi: 10.1007/s10389-023-02012-1

[5] Hua BH Sugumaran SV Faryza E Atiqah N Jasvindar K Kabir MS . Prevalence of musculoskeletal disorders (MSD) and smartphone addictions among university students in Malaysia. Int J Health Sci. (2022) 6:1075–88. doi: 10.53730/ijhs.v6nS2.5078

[6] Sirajudeen MS Alzhrani M Alanazi A Alqahtani M Waly M Manzar MD . Prevalence of upper limb musculoskeletal disorders and their association with smartphone addiction and smartphone usage among university students in the Kingdom of Saudi Arabia during the COVID 19 pandemic—a cross-sectional study. Healthcare (2022) 10:2373. doi: 10.3390/healthcare10122373 36553897 PMC9777717

[7] Choi Y Yang X Park J Lee W You H. Effects of smartphone size and hand size on grip posture in one-handed hard key operations. Appl Sci. (2020) 10:8374. doi: 10.3390/app10238374

[8] Amjad F Farooq MN Batool R Irshad A. Frequency of wrist pain and its associated risk factors in students using mobile phones. Pak J Med Sci. (2020) 36:746–9. doi: 10.12669/pjms.36.4.1797 32494267 PMC7260896

[9] Mustafaoglu R Yasaci Z Zirek E Griffiths MD Ozdincler AR. The relationship between smartphone addiction and musculoskeletal pain prevalence among young population: a cross-sectional study. Korean J Pain (2021) 34:72–81. doi: 10.3344/kjp.2021.34.1.72 33380570 PMC7783853

[10] Bhamra JK Naqvi WM Arora SP. Effect of smartphone on hand performance and strength in the healthy population. Cureus (2021) 13:e15798. doi: 10.7759/cureus.15798 34306866 PMC8294013

[11] Alshahrani A Samy Abdrabo M Aly SM Alshahrani MS Alqhtani RS Asiri F . Effect of smartphone usage on neck muscle endurance, hand grip and pinch strength among healthy college students: a cross-sectional study. Int J Environ Res Public Health (2021) 18:6290. doi: 10.3390/ijerph18126290 34200762 PMC8296110

[12] de Jesus Correia F Soares JB Dos Anjos Matos R Pithon KR Ferreira LN de Assunção PL. Smartphone addiction, musculoskeletal pain and functionality in university students - a observational study. Psychol Health Med. (2024) 29:286–96. doi: 10.1080/13548506.2023.2176893 36803275

[13] Guedes-Aguiar ED Taiar R Paineiras-Domingos LL Monteiro-Oliveira BB da Cunha de Sá-Caputo D Bernardo-Filho M. Effects of a single session of systemic vibratory therapy on flexibility, perception of exertion and handgrip strength in chronic obstructive pulmonary disease individuals: a quasi-experimental clinical trial. J Clin Med. (2023) 12:3241. doi: 10.3390/jcm12093241 37176687 PMC10179630

[14] Cristino De Souza AL Mendonça VA Coelho De Oliveira AC Ferreira Da Fonseca S Mello Santos LM Cunha Fernandes JS . Whole body vibration in the static modified push-up position in untrained healthy women stimulates neuromuscular system potentiating increased handgrip myogenic response. J Bodyw Mov Ther. (2020) 24:233–8. doi: 10.1016/j.jbmt.2020.06.021 33218516

[15] Sharma S Hayat Z. Effects of whole-body vibration on sports performance: a systematic review and meta-analysis. Sci Sports (2022) 37:231–43. doi: 10.1016/j.scispo.2021.06.015

[16] Coelho-Oliveira AC Lacerda ACR De Souza ALC Santos LMDM Da Fonseca SF Dos Santos JM . Acute whole-body vibration exercise promotes favorable handgrip neuromuscular modifications in rheumatoid arthritis: a cross-over randomized clinical. BioMed Res Int. (2021) 2021:9774980. doi: 10.1155/2021/9774980 34901282 PMC8660187

[17] Zeng D Zhao K Lei W Yu Y Li W Kong Y . Effects of whole-body vibration training on physical function, activities of daily living, and quality of life in patients with stroke: a systematic review and meta-analysis. Front Physiol. (2024) 15:1295776. doi: 10.3389/fphys.2024.1295776 38322612 PMC10844406

[18] Santos LMM Oliveira ACC Fonseca SF Silva AF Santos JNV Souza ALC . Whole-body vibration exercise in different postures on handgrip strength in healthy women: a cross-over study. Front Physiol. (2021) 11:469499. doi: 10.3389/fphys.2020.469499 33536927 PMC7848817

[19] Jo NG Kang SR Ko MH Yoon JY Kim HS Han KS . Effectiveness of whole-body vibration training to improve muscle strength and physical performance in older adults: prospective, single-blinded, randomized controlled trial. Healthcare (2021) 9:652. doi: 10.3390/healthcare9060652 34072657 PMC8226869

[20] Alghadir AH Gabr SA Rizk AA Alghadir T Alghadir F Iqbal A. Smartphone addiction and musculoskeletal associated disorders in university students: biomechanical measures and questionnaire survey analysis. Eur J Med Res. (2025) 30:274. doi: 10.1186/s40001-025-02413-w 40229835 PMC11998459

[21] Petrie KA Burbank K Sizer PS James CR Zumwalt M. Considerations of sex differences in musculoskeletal anatomy between males and females. In: The Active Female: Health Issues Throughout the Lifespan. Cham: Springer International Publishing (2023). p. 3–24. doi: 10.1007/978-3-031-15485-0_1

[22] Al-Qarni MS El Keshky ME. Psychometric properties of the Arabic short version of the smartphone addiction scale (SAS-SV) in Saudi Arabia. J King Abdulaziz Univ Arts Humanit. (2022) 30:401–14. doi: 10.4197/Art.30-2.14

[23] Banadaki FD Rahimian B Moraveji F Varmazyar S. The impact of smartphone use duration and posture on the prevalence of hand pain among college students. BMC Musculoskelet Disord. (2024) 25:574. doi: 10.1186/s12891-024-07685-7 39044247 PMC11265474

[24] Hosokawa T Tajika T Suto M Chikuda H. The quick disabilities of the arm, shoulder, and hand (QuickDASH) scores in 961 Japanese volunteers. J Orthop Surg. (2020) 28:2309499020970656. doi: 10.1177/2309499020970656 33169638

[25] Perchthaler D Hauser S Heitkamp HC Hein T Grau S. Acute effects of whole-body vibration on trunk and neck muscle activity in consideration of different vibration loads. J Sports Sci Med. (2015) 14:155–62. 25729303 PMC4306767

[26] Ahn JY Kim H Park CB. Effects of whole-body vibration on upper extremity function and grip strength in patients with subacute stroke: a randomised single-blind controlled trial. Occup Ther Int. (2019) 2019:5820952. doi: 10.1155/2019/5820952 31065236 PMC6466864

[27] Aksoy D. Effects of 10-week whole body vibration training on strength, flexibility and agility in taekwondo athletes. J Educ Learn. (2019) 8:213–22. doi: 10.5539/jel.v8n2p213

[28] Di Giminiani R Fabiani L Baldini G Cardelli G Giovannelli A Tihanyi J. Hormonal and neuromuscular responses to mechanical vibration applied to upper extremity muscles. PLoS ONE (2014) 9:e111521. doi: 10.1371/journal.pone.0111521 25368995 PMC4219718

[29] Abbas R El Khatib A M. Saab I. Effect of segmental vibration on hand and pinch grip strengths in healthy subjects. BAU J Health Wellbeing (2020) 2:1034. doi: 10.54729/2789-8288.1034

[30] Vinstrup J Calatayud J Jakobsen MD Sundstrup E Jørgensen JR Casaña J . Hand strengthening exercises in chronic stroke patients: dose-response evaluation using electromyography. J Hand Ther. (2018) 31:111–21. doi: 10.1016/j.jht.2017.01.004 28527751

[31] Plant CE Parsons NR Edwards AT Rice H Denninson K Costa ML . comparison of electronic and manual dynamometry and goniometry in patients with fracture of the distal radius and healthy participants. J Hand Ther. (2016) 29:73–80. doi: 10.1016/j.jht.2015.11.004 26847323

[32] Stamate A Bertolaccini J Deriaz M Gunjan S Marzan MD Spiru L. Interinstrument reliability between the Squegg^®^ smart dynamometer and hand grip trainer and the Jamar^®^ hydraulic hand dynamometer: a pilot study. Am J Occup Ther. (2023) 77:7705205150. doi: 10.5014/ajot.2023.050099 37824723

[33] Rajendiran S Pai GM Verma V Rajappa S Bhat A Gaba S . Normative data of grip strength and pinch strength in the Indian population. Indian J Plast Surg. (2024) 57:256–62. doi: 10.1055/s-0044-1788999 39345673 PMC11436323

[34] Forman DA Forman GN Holmes MWR. Wrist extensor muscle activity is less task-dependent than wrist flexor muscle activity while simultaneously performing moderate-to-high handgrip and wrist forces. Ergonomics (2021) 64:1595–605. doi: 10.1080/00140139.2021.1934564 34024262

[35] Zhao Y Li Z Zhang Z Qian K Xie S. An EMG-driven musculoskeletal model for estimation of wrist kinematics using mirrored bilateral movement. Biomed Signal Process Control (2023) 81:104480. doi: 10.1016/j.bspc.2022.104480

[36] Alnahdi AH. Validity and reliability of the Arabic quick disabilities of the arm, shoulder and hand (QuickDASH-Arabic). Musculoskelet Sci Pract. (2021) 53:102372. doi: 10.1016/j.msksp.2021.102372 33780697

[37] Villafañe JH Valdes K Bertozzi L Negrini S. Minimal clinically important difference of grip and pinch strength in women with thumb carpometacarpal osteoarthritis when compared to healthy subjects. Rehabil Nurs. (2017) 42:139–45. doi: 10.1002/rnj.196 25557054

[38] Heneidy WE Farouk MA Olama KA Ibrahim AF. Effect of vibration therapy on upper limb range of motion and muscle strength in children with hemiplegia: a randomized controlled trial. Bull Fac Phys Ther. (2025) 30:12. doi: 10.1186/s43161-025-00274-2

[39] Li W Li Y Wang Z Lu Y Qiu Y Li Z . Effects of 12 weeks of whole-body vibration training and vitamin D supplementation on bone density and muscle quality in the aged with osteosarcopenia: a randomized controlled trial. Gerontology (2025) 71:899–909. doi: 10.1159/000547822 40971339

[40] Ni CH Lu YH Chou LW Kuo SF Lin CH Chiang SL . Analysis of vibration frequency and direction for facilitating upper-limb muscle activity. Biology (2022) 12:48. doi: 10.3390/biology12010048 36671741 PMC9855852

[41] Monteiro-Oliveira BB Coelho-Oliveira AC Paineiras-Domingos LL Sonza A Sá-Caputo DD Bernardo-Filho M. Use of surface electromyography to evaluate effects of whole-body vibration exercises on neuromuscular activation and muscle strength in the elderly: a systematic review. Disabil Rehabil. (2022) 44:7368–77. doi: 10.1080/09638288.2021.1994030 34699285

[42] Liu Y Fan Y Chen X. Effects of whole-body vibration training with different body positions and amplitudes on lower limb muscle activity in middle-aged and older women. Dose Response (2022) 20:15593258221112960. doi: 10.1177/15593258221112960 35859854 PMC9289914

[43] Zhang J Wang R Zheng Y Xu J Wu Y Wang X. Effect of whole-body vibration training on muscle activation for individuals with knee osteoarthritis. Biomed Res Int. (2021) 2021:6671390. doi: 10.1155/2021/6671390 33855078 PMC8019384

[44] Yoon JY Kang SR Kim HS Won YH Park SH Seo JH . Effects of low-frequency whole-body vibration on muscle activation, fatigue, and oxygen consumption in healthy young adults: a single-group repeated-measures controlled trial. Sport Rehabil. (2022) 31:984–92. doi: 10.1123/jsr.2021-0170 35584804

[45] Galardini L Coppari A Pellicciari L Ugolini A Piscitelli D La Porta F . Minimal clinically important difference of the disabilities of the arm, shoulder and hand (DASH) and the shortened version of the DASH (QuickDASH) in people with musculoskeletal disorders: a systematic review and meta-analysis. Phys Ther. (2024) 104:pzae033. doi: 10.1093/ptj/pzae033 38438144 PMC11997799

[46] Alam MM Khan AA Farooq M. Effects of vibratory massage therapy on grip strength, endurance time and forearm muscle performance. Work (2021) 68:619–32. doi: 10.3233/WOR-203397 33612507

[47] Lu YH Chen HJ Liao CD Chen PJ Wang XM Yu CH . Upper extremity function and disability recovery with vibration therapy after stroke: a systematic review and meta-analysis of RCTs. J NeuroEng Rehabil. (2024) 21:221. doi: 10.1186/s12984-024-01515-6 39707380 PMC11662454

[48] Lee JS Kim CY Kim HD. Short-term effects of whole-body vibration combined with task-related training on upper extremity function, spasticity, and grip strength in subjects with poststroke hemiplegia: a pilot randomized controlled trial. Am J Phys Med Rehabil. (2016) 95:608–17. doi: 10.1097/PHM.0000000000000454 26829094

[49] Kelaiditi M Lepoura A Christakou A Chrysagis N Sakellari V. The effect of whole-body vibration on upper extremity function in children with cerebral palsy: a pilot study. Appl Sci. (2025) 15:552. doi: 10.3390/app15020552

